# Prospective and Multicenter Evaluation of Outcomes for Quality of Life and Activities of Daily Living for Balloon Kyphoplasty in the Treatment of Vertebral Compression Fractures: The EVOLVE Trial

**DOI:** 10.1093/neuros/nyy017

**Published:** 2018-03-14

**Authors:** Douglas P Beall, M R Chambers, Sam Thomas, John Amburgy, James R Webb, Bradly S Goodman, Devin K Datta, Richard W Easton, Douglas Linville, Sanjay Talati, John B Tillman

**Affiliations:** 1 Department of Radiology, Clinical Radiology of Oklahoma, Edmond, Oklahoma; 2 Interventional Spine Services, The Spine Fracture Institute, Edmond, Oklahoma; 3 Department of Neurological Surgery, University of Alabama at Birmingham, Birmingham, Alabama; 4 Allegheny College, Meadville, Pennsylvania; 5 Dr James Webb & Associates’ Osteoporosis Institute, Tulsa, Oklahoma; 6 Alabama Clinical Therapeutics, LLC, Birmingham, Alabama; 7 Alabama Ortho Spine and Sports, Birmingham, Alabama; 8 The Back Center, Melbourne, Florida; 9 Beaumont Health System, Troy, Michigan; 10 Scoliosis & Spine Surgery Clinic of Memphis, PLLC, Memphis, Tennessee; 11 Advanced Diagnostic Imaging, PC, Saginaw, Michigan; 12 Medtronic Inc, Clinical Program, Milpitas, California

**Keywords:** Back pain, Balloon kyphoplasty, Neoplastic fractures, Osteoporosis, Quality of life, Vertebral augmentation, Vertebral compression fracture

## Abstract

**BACKGROUND:**

Osteoporotic and neoplastic vertebral compression fractures (VCF) are common and painful, threatening quality of life and increasing risk of morbidity and mortality. Balloon kyphoplasty is a percutaneous option for treating painful cancer- and osteoporosis-related VCFs, supported by 2 randomized trials demonstrating efficacy benefits of BKP over nonsurgical care.

**OBJECTIVE:**

To investigate 12-mo disability, quality of life, and safety outcomes specifically in a Medicare-eligible population, representing characteristic patients seen in routine clinical practice.

**METHODS:**

A total of 354 patients with painful VCFs were enrolled at 24 US sites with 350 undergoing kyphoplasty. Four coprimary endpoints—Numerical Rating Scale (NRS) back pain, Oswestry Disability Index (ODI), Short Form-36 Questionnaire Physical Component Summary (SF-36v2 PCS), EuroQol-5-Domain (EQ-5D)—were evaluated for statistically significant improvement 3 mo after kyphoplasty. Data were collected at baseline, 7 d, and 1, 3, 6, and 12 mo (www.clinicaltrials.gov registration NCT01871519).

**RESULTS:**

At the 3-mo primary endpoint, NRS improved from 8.7 to 2.7 and ODI improved from 63.4 to 27.1; SF-36 PCS was 24.2 at baseline improving to 36.6, and EQ-5D improved from 0.383 to 0.746 (*P* < .001 for each). These outcomes were statistically significant at every follow-up time point. Five device-/procedure-related adverse events, intraoperative asymptomatic balloon rupture, rib pain, and aspiration pneumonia, and a new VCF 25 d postprocedure, and myocardial infarction 105 d postprocedure were reported and each resolved with proper treatment.

**CONCLUSION:**

This large, prospective, clinical study demonstrates that kyphoplasty is a safe, effective, and durable procedure for treating patients with painful VCF due to osteoporosis or cancer.

**ABBREVIATIONS**
AE: adverse event
CI: confidence interval
EDC: electronic data capture
EQ-5D: EuroQol-5-Domain
IBT: inflatable bone tamp
MCID: minimally clinically important difference
MR: magnetic resonance
NSM: nonsurgical management
ODI: Oswestry Disability Index
PCS: physical component summary
RCT: randomized control trial
SAE: serious adverse event
SF-36: short form-36
VBA: vertebral body angulation
VCF: vertebral compression fracture

The clinical significance of vertebral compression fractures (VCF) is severe physical limitation, disability, and increased morbidity and mortality^1-6^ with considerable associated annual heath care expenditures.^7^

Osteoporosis is the most common condition associated with VCF; worldwide, VCFs affect30% to 50% of people over 50 yr of age. Vertebral fracture incidence increases substantially with age in both males and females.^8^ The presence of vertebral fracture is associated with increased risk of future fractures.^9^ Additionally, in cancer, risk for pathologic VCF can arise due to bone metastases, estimated to be 24%, 14%, 6%, and 8% among patients with multiple myeloma and cancers of the breast, prostate, and lung, respectively.^10^

Chen et al^1^ found significant reductions in hospitalization time and mortality in patients treated with vertebral augmentation as compared with those patients treated with nonsurgical management (NSM); a more recent study showed slightly longer hospitalization but greater discharge to home for augmented patients.^6^ Recent Medicare claims-based analyses of over 1000 000 VCF patients with 5 to 10 yr follow-up, performed with propensity score matching to account for selection bias, concluded that there was a highly statistically significant reduction of both morbidity and mortality in patients treated with vertebral augmentation as compared to those treated with NSM.^3,6^ In these analyses comparing NSM to vertebral augmentation, NSM patients had significantly higher rates of pneumonia, deep venous thrombosis, cardiac complications, and urinary tract infections.^3,6^

Kyphoplasty proved superior to NSM in randomized control trials (RCT), improving pain, function, quality of life and patient satisfaction.^11-14^ Notwithstanding this level I evidence, there remains a paucity of clinical trial evidence showing the benefits of Balloon Kyphoplasty (BKP) in a typical on-label, as-treated patient population involving consecutive patients meeting common inclusion/exclusion criteria.

## METHODS

### Study Design and Patients

A prospective, phase IV, open-label, multicenter, 12-mo clinical study was conducted; outcomes included activities of daily living, pain, quality of life, and safety parameters in a Medicare-eligible population treated with kyphoplasty for painful acute or subacute VCFs associated with osteoporosis or cancer. The protocol and informed consent were approved by the institutional review board at each study center. All patients provided written informed consent. A total of 354 patients were enrolled at 24 sites between May 2013 and October 2014; last patient, last visit was completed on December 2015. In preparing this manuscript STROBE recommendations were followed (http://www.strobe-statement.org).

Medicare-eligible patients with 1 to 3 painful VCFs from T5 to L5 due to osteoporosis or cancer, with clinical findings (pain on palpation or percussion over the fractured vertebral body) correlating with imaging findings, were eligible. Acute or subacute (≤ 4 mo) status of fracture(s) were based on magnetic resonance (MR) or nuclear bone scan or an acute change in VB height or morphology from a previous x-ray, computed tomography, or MR. Pretreatment Numerical Rating Scale (NRS) score ≥ 7 and Oswestry Disability Index (ODI) score ≥ 30, and mental capacity to comply with protocol requirements for the 12-mo study duration were required. Subjects with vertebral morphology or fracture configuration contraindicating BKP (eg, split fracture, complete burst fracture, pedicle fracture), VCFs due to high-energy trauma, asymptomatic VCFs, VCF accompanied by objective evidence of neurological compromise, spinal cord compression or canal compromise requiring decompression were excluded as were patients with pre-existing conditions or clinical comorbidities contraindicating surgery or precluding long-term follow-up. The trial is registered, and a complete list of inclusion/exclusion criteria is posted on www.clinicaltrials.gov (NCT01871519). Bone mineral density measurements were made by dual axial absorptiometry scanning but were not required, as low-energy VCF supports the diagnosis of osteoporosis.^9,15^

### Procedures

Three hundred fifty patients underwent kyphoplasty in standard fashion per manufacturer's instructions. Briefly, cannulae are placed through either 1 or both pedicles (or alternatively extrapedicularly) using fluoroscopic guidance. An inflatable bone tamp (IBT) is inserted through each cannula into the vertebral body and inflated using radiopaque contrast and a pressure-measuring device (Kyphon Xpander I or II IBTs, Medtronic, Memphis, Tennessee). Balloon inflation is stopped once maximum pressure/volume is reached, desired fracture reduction is achieved or if balloons reach cortical walls or there are any signs of cortical breach. The IBT(s) is deflated, removed, and the void created within the vertebral body is filled with viscous polymethylmethacrylate (Kyphon HV-R or Xpede, Medtronic, Memphis, Tennessee) bone cement.^11-14^

### Outcomes

The primary objective was to show statistically significant improvement from baseline in 4 coprimary endpoints at 3 mo: back pain NRS (scale 0-10), ODI (scale 0-100), Short Form-36 Questionnaire Physical Component Summary (SF-36v2 PCS, scale 0-100), and EuroQol-5-Domain (EQ-5D, scale 0-1). As previously described, each subject completed these pre- and postoperative questionnaires in person at each clinic visit (NRS was also collected by phone at 7 d).^16^ Subjects were asked to complete these questionnaires a second time at baseline but instructed to do so as if prior to their fracture, in order to collect estimated back pain, disability, and quality of life before the incident VCF.^17^ Following the procedure, 4 coprimary endpoints and secondary outcomes were assessed at 1, 3, 6, and 12 mo. Secondary outcomes included ambulatory status,^18^ procedure information, medication usage, bed rest and limited activity days, kyphotic angulation correction, adverse events (AE), cement leakage, and new vertebral fractures.^11-14,16^ Subjects with osteoporosis completed the Barthel Index,^19^ and physicians assessed cancer patients using the Karnofsky Performance Scale as previously described.^14^ Cement leakage was determined by investigator review of intraoperative fluoroscopy and included any cement outside the vertebral borders.^12,13,18^

Standing lateral spine radiographs were taken at baseline, immediately postoperatively and at 3 and 12 mo, to assess for new fractures using the method of Genant according to methods previously described^13^; vertebral body angulation (VBA) and vertebral body height were evaluated using quantitative morphometry as previously described.^12^ All images were read centrally (BioClinica, Newark, California) by an independent radiologist.

All AEs were collected, reported, and evaluated by investigators for device- and procedure-relationship. The AEs were systematically classified into preferred terms and system organ class according to the Medical Dictionary for Regulatory Activities using the verbatim language reported by investigators into the electronic data capture (EDC) system provided (ICON, North Wales, Pennsylvania).^16^ For medication usage, sites recorded verbatim drug names, indication for use, along with start and stop dates. Verbatim drug names were reviewed and systematically coded according to the World Health Organization drug dictionary. The number of subjects taking pain medications within each classification (eg, opioids, non-steroidal anti-inflammatories, muscle relaxants) were reported according to visits.

### Statistical Power

In order to have a minimum power of 80%, or β = 0.80 with 4 coprimary variables, each of the 4 variables must have β = 0.80^1/4^ or 0.95. Conservatively, assuming no correlation of the 4 coprimary endpoints, a 5% alpha, and standard deviations of 11, 3.3, 20, and 0.27^16^ for SF-36v2 PCS, NRS, ODI, and EQ-5D, respectively, 300 subjects would provide >95% power for each outcome, using a 1-sided paired *t*-test, to detect a minimal difference of 2.1, 0.63, 3.81, and 0.052, respectively. These values are well below the minimally clinically important differences (MCIDs) for these parameters.^20-23^ Anticipating 14% loss to follow-up at 3 mo,^11^ 350 treated subjects were required to show statistically significant change in each of the coprimary outcomes. Given the parameters above, the overall study power was 82%.

### Statistical Analysis

Analyses of demographic, surgical, ambulatory status, and safety variables were descriptive in nature. One-sided paired *t*-tests were used for each of 4 coprimary endpoints to assess whether the mean change from baseline at 3 mo was significantly <0 (NRS, ODI) or >0 (SF-36v2 PCS, EQ-5D), if the endpoints satisfied the assumption of normality (assessed by using q-q plots and the Shapiro–Wilk test); if not, analysis was performed using the Wilcoxon signed-rank test; there was no imputation for missing data. If the *P*-value from each test for each endpoint was ≤.05, then the primary objective was met.

Similarly, for secondary endpoints, a *P*-value was provided for the comparison between follow-up and baseline to see whether the change was significantly improved; no adjustments were made. A paired t-test for normal data or Wilcoxon signed-rank test for non-normal data was used to produce *P*-values.

## RESULTS

### Participants

Three hundred fifty-four subjects were enrolled; 4 subjects withdrew prior to receiving treatment; within the 350 subjects treated, 7 subjects were included who had deviations to the inclusion/exclusion criteria (1 was not Medicare eligible; 1 had a fracture age slightly >4 mo, 4 had NRS scores < 7, and 1 was participating in another clinical study). Forty-nine patients voluntarily withdrew prior to the 12-mo assessment, 15 were lost to follow-up, and 26 deaths occurred from causes unrelated to treatment. One patient who terminated the study early experienced an AE (a decline in general health leading to hospice care), precluding continued participation. Two hundred sixty patients completed the study (Table 1).

**TABLE 1.** Subject Accountability

	Baseline	Surgery	7 d	30 d	3 mo	6 mo	9 mo	12 mo
Subjects enrolled	354							
Subjects not treated and withdrew	4							
Subjects enrolled and treated		350						
Cumulative deaths		0	0	3	10	14	21	26
Cumulative withdrawals		0	2	10	25	34	36	49
Cumulative lost follow-up		0	0	0	4	7	10	15
Expected visit		350	348	337	311	295	283	260
Subjects followed with data		350	348	324	302	273	280	260
Follow-up rate of expected (%)		100	100	96.1	97.1	92.5	98.9	100

### Descriptive Data

The average age was 78.9 yr, 78.0% were female and 36.7% had a clinical history of prior fractures (Table 2). Eight of 354 subjects (2.3%) had fracture etiology due to cancer at baseline.

**TABLE 2.** Subject Characteristics

Variable	BKP (n = 354)
Age, mean (range)	78.9 (51-100)
Female, n (%)	276 (78.0)
Body mass index, mean (range)	26.1 (12.6-43.7)
Caucasian, n (%)	333 (94.1)
Smoking, n (%)	
Never	195 (55.1)
Former	131 (37.0)
Current	28 (7.9)
Working prior to VCF, n (%)	36 (10.2)
Working after VCF, n (%)	18 (5.1)
Estimated fracture age, mean (Standard Deviation (SD))	34.7 (27.8)
Etiology of VCF, n (%)	
1º osteoporosis	316 (89.3)
2º osteoporosis	30 (8.5)
Cancer	8 (2.3)
Subjects with any prior fracture, n (%)	
Yes	130 (36.7)
No	224 (63.3)

**FIGURE 1.**
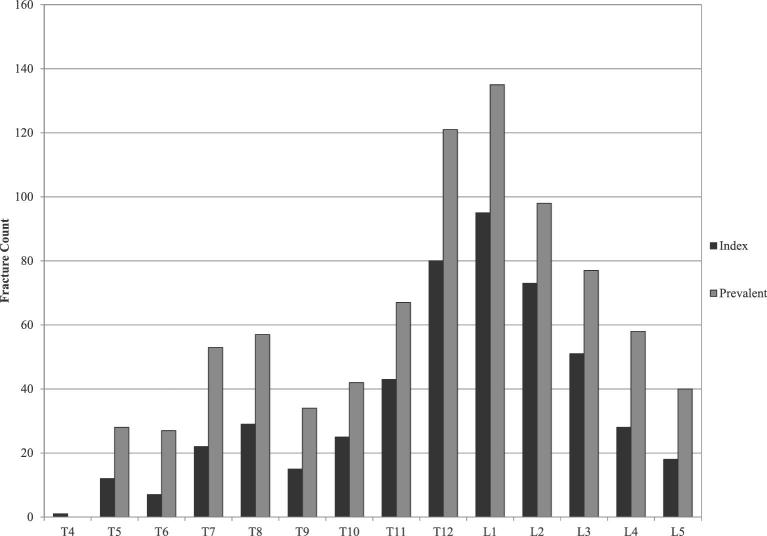
Distribution of index and prevalent fracture levels. Index levels (those identified by investigators as treatment levels) and prevalent fractures (all radiographic fractures assessed by the core laboratory) are shown; prevalent fractures were identified from standing lateral x-ray films with 344 of 350 treated patients contributing data.

Three hundred fifty subjects were treated at 499 levels; 64.3% had single fractures treated (Table 3). Bilateral kyphoplasty was performed in 54.9% of levels. Most subjects underwent local anesthesia with conscious sedation (67.1%) and most were treated as outpatients (79.1%). Mean procedure duration was 24.4 min; mean fluoroscopy duration was 5.0 min and mean length of stay was 9.2 h. One hundred fifty-eight of 350 patients had a biopsy. One hundred fifty patients had confirmatory negative findings, 1 had findings of devitalized bone, and 3 had inconclusive findings; 2 subjects initially diagnosed with osteoporosis had cancer findings and 2 cancer subjects had confirmatory findings. Asymptomatic cement leakage was reported in 107/499 (21.4%) index levels treated (Table 3). The majority of leaks were into adjacent disc spaces or paraspinal tissue (86/107; 80.4%). Approximately 70% of fractures treated were from T10 to L3, and there was higher radiographic fracture prevalence than those identified clinically (Figure 1).

**TABLE 3.** Procedure Characteristics

Variable	BKP (n = 350)
Location of procedure, n (%)	
Hospital	254 (72.6)
Ambulatory Surgery Center	28 (8.0)
Office	68 (19.4)
Hospitalization, n (%)	
Inpatient	73 (20.9)
Outpatient	277 (79.1)
Anesthesia, n (%)	
General	115 (32.9)
Local	235 (67.1)
Procedure duration in min, mean (SD)	24.4 (12.4)
Fluoroscopy duration in min, mean (SD)	5.0 (5.3)
Length of stay, mean in h (SD)	9.2 (16.1)
#VCF treated, n (%)	
1	225 (64.3)
2	101 (28.9)
3	24 (6.9)
VCF treated	499
Procedure, n (%)	
Unilateral	225 (45.1)
Bilateral	274 (54.9)
Cement volume in cc, mean (SD)	5.3 (2.2)
Balloon volume in cc, mean (SD)	
Right	2.6 (1.0)
Left	2.6 (1.1)
Balloon pressure in PSI, mean (SD)	
Right	195.4 (94.8)
Left	181.7 (96.4)
Cement leakage present, n (%)	107 (21.4)
Superior disc	29
Inferior disc	23
Epidural space	12
Foraminal space	1
Paraspinal tissue	34
Intravascular	8
Extruded ≥ 15 mm	0

### Main Results

**FIGURE 2.**
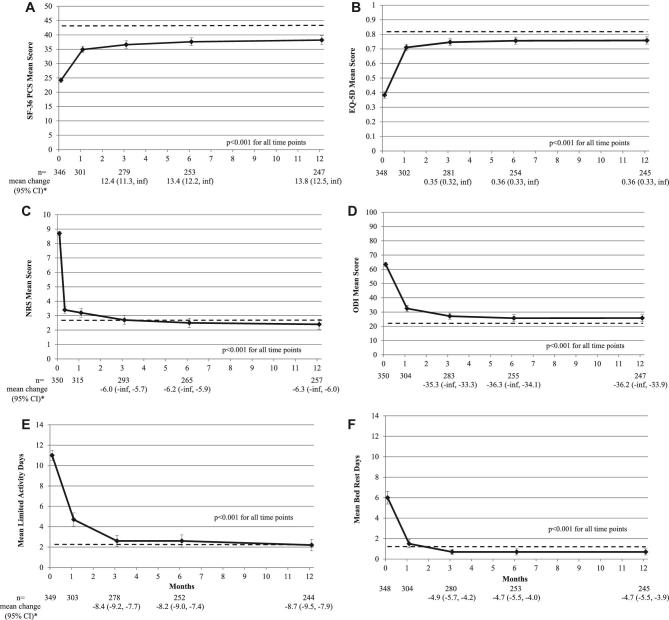
Quality of life, disability, and pain assessments at baseline and after balloon kyphoplasty. Raw mean scores and 95% confidence intervals (CIs) are shown as ‘error bars’ for balloon kyphoplasty (solid lines) for **A**, SF-36 PCS (scale 0-100); **B**, total EQ-5D scores (scale 0-1); **C**, back pain (scale 0-10); **D**, ODI (scale 0-100); **E**, limited activity days (scale 0-14); **F**, bed rest days (scale 0-14). The *P*-value in each panel is for all postoperative visits. Below each panel, the n for each group is shown for baseline, 3, 6, and 12 mo as well as the group average for change from baseline and 95% CI (in parentheses) for 3, 6, and 12 mo. Please note that for the coprimary endpoints in panels **A**–**D**, the 95% CI are 1-sided, reflecting the critical lower bound while the upper bound is infinity. The 95% CI reflected in panels **E** and **F** are 2-sided. The dashed line indicates the average prefracture estimation from EVOLVE patients for that parameter.

There was statistically significant improvement from baseline in each of the 4 coprimary endpoints at 3 mo. NRS back pain average baseline score of 8.7 improved 6.0 points. For ODI, average baseline score of 63.4 improved 35.3 points (*P* < .001 for each). Similarly, SF-36v2 PCS average baseline score of 24.2 points improved 12.4 points, and EQ-5D average baseline score of 0.383 improved 0.351 points (*P* < .001 for each). Statistically significant improvement was observed at all time points for these outcomes, in addition to improvements in limited activity and bed rest days (Figure 2). Within 1 to 3 mo after BKP treatment, patients have outcomes that are close to those estimated prior to the fracture event (Figure 2). Because few cancer subjects were enrolled in the study, as a secondary analysis, we analyzed the coprimary endpoints in osteoporosis subjects only and found nearly identical results (data not shown).

### Outcome Data

As a sensitivity analysis, the coprimary outcome measures from subjects enrolled from investigators receiving consultancy payments compared to subjects enrolled by investigators without these potential conflicts were evaluated. Back pain and ODI scores had some time points with statistically significant greater improvement (SF-36 PCS and EQ-5D were not statistically significant at any time point) in a single center where the investigator had received consultancy payments (data not shown). For example, NRS scores improved from an average of 9.0 to 1.4 at 12 mo at this center vs an average of 8.6 to 2.5 in other centers (*P* = .038). It should be noted that at baseline, patients at the single center had estimated a lower prefracture pain state at baseline (average of 1.7 points vs 2.8; *P* = .028), and therefore in either cohort, on average, 12-mo pain scores were close to the estimated pain prior to the fracture event.

Subjects taking opioid analgesics decreased from 71.6% at baseline to 54.9% at 12 mo; the number of subjects requiring back bracing, bed rest, and limited activity was also substantially reduced over time (see Table 4).

**TABLE 4.** Nonsurgical Treatments Received

Variable	Baseline	3 mo	12 mo
Any pain medications	256/303 (84.5%)	207/245 (84.5%)	151/184 (82.1%)
Opioids	217/303 (71.6%)	159/245 (64.9%)	101/184 (54.9%)
Muscle relaxants	36/303 (11.9%)	28/245 (11.4%)	17/184 (9.2%)
Non-steroidal anti-inflammatories	38/303 (12.5%)	33/245 (13.5%)	31/184 (16.8%)
Other analgesics/antipyretics	62/303 (20.5%)	65/245 (26.5%)	55/184 (29.9%)
Nonsurgical care	176/350 (50.3%)	68/302 (22.5%)	47/260 (18.1)
Bed rest	71/350 (20.3%)	3/302 (1.0%)	1/260 (0.4%)
Back bracing	67/350 (19.1%)	5/302 (1.7%)	3/260 (1.2%)
Walking aids	35/350 (10.0%)	16/302 (5.3%)	17/260 (6.5%)
Wheelchair	10/350 (2.9%)	0/302 (0%)	0/260 (0%)
Physical therapy	18/350 (5.1%)	32/302 (10.6)	12/260 (4.6%)
Pain management program	14/350 (4.0%)	3/302 (1.0%)	7/260 (2.7%)
Limit activity	337/350 (96.3%)	90/283 (31.8%)	71/246 (28.9%)

Improvement in Barthel Index was statistically significant at each follow-up; improvements in Karnofsky were not statistically significant likely due to the small number of cancer subjects enrolled (Table 5). Ambulatory status was improved with 42.3% of subjects able to walk without assistance at baseline increasing to 63.2% at 12 mo.

**TABLE 5.** Performance and Ambulatory Status

		Baseline	1 mo		3 mo		6 mo		12 mo	
Outcome measure		BKP	BKP	*P*-value	BKP	*P*-value	BKP	*P*-value	BKP	*P*-value
Barthel Index (scale 0-20)^a^		n = 343	n = 299	<.001	n = 280	<.001	n = 253	<.001	n = 244	<.001
		16.2	18.8		19.1		19.1		19.1	
		(15.8, 16.6)	(18.5, 19.0)		(18.8, 19.3)		(18.9, 19.3)		(18.9, 19.4)	
Karnofsky performance scale (0-100)^b^		n = 7	n = 5	.3	n = 4	.5	n = 4	1.0	n = 3	1.0
		75.7	88.0		90.0		85.0		96.7	
		(55.8, 95.6)	(74.4, 101.6)		(67.5, 112.5)		(37.3, 132.7)		(82.3, 111.0)	
Ambulatory status^c^										
	Walk without assistance	148/350 (42.3)	197/315 (62.5)	ND	189/293 (64.5)	ND	166/266 (62.4)	ND	163/258 (63.2)	ND
	Walk with aid	176/350 (50.3)	114/315 (36.2)		100/293 (34.1)		96/266 (36.1)		92/258 (35.7)	
	Unable to walk	26/350 (7.4)	4/315 (1.3)		4/293 (1.4)		4/266 (1.5)		3/258 (1.2)	

^a^Barthel Index was only collected in osteoporosis subjects; table reflects mean (95% CI).

^b^Karnofsky performance was only collected in cancer subjects; table reflects mean (95% CI).

^c^Ambulatory status was collected in all subjects; table reflects numerator/denominator (%); ND indicates statistical change from baseline was not done.

**FIGURE 3.**
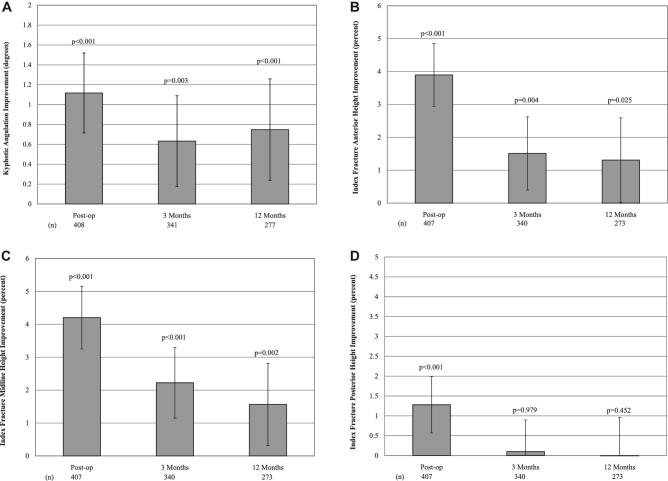
Index vertebral body kyphotic angulation correction and height restoration. Means and 95% CIs are shown for **A**, kyphotic angulation of index fractures; **B**, index fracture anterior height as a percent; **C**, index fracture midvertebral height as a percent; **D**, index fracture posterior height as a percent. *p*-values for change from baseline improvement are shown for each time point. Below each panel, the n for each group is shown for baseline, 3, and 12 mo.

The mean VBA was –10.5^o^ at baseline in 490 treated levels with evaluable VBA data. Improvement was statistically significant at predischarge (1.117º, *P* < .001), 3 mo (0.633º, *P* = .003), and 12 mo (0.748º, *P* < .001). Similar observations were made in anterior- and mid-vertebral height restoration (Figure 3).

At 3 mo postoperatively, there were 98 of 267 (36.7%) patients with subsequent fractures. This number increased to 117 of 246 patients (47.6%) at 12 mo. Of those, 63 of the 267 (23.6%) and 72 of the 246 subjects (29.3%) had fractures adjacent to a treated level.

### Safety

The most common serious adverse events (SAEs) within 30 d of surgery were back pain (14/350 or 4.0%) and new symptomatic fracture (5/350 or 1.4%; Table 6). These were also the most common SAE categories over 1 yr of follow-up: back pain (17/350 or 4.9%) and new symptomatic fracture (16/350 or 4.6%). Five AEs that were possibly device- or procedure-related included an asymptomatic balloon rupture considered cement-related and another subject with rib pain considered possibly cement-related that began intraoperatively and resolved within 6 mo; another subject had a new adjacent VCF AE 25 d postprocedure considered possibly cement-related. None of these patients with cement-related AEs were reported to have any cement leakage.

**TABLE 6.** Adverse Events Within 30 d of Procedure

	Kyphoplasty
Number of patients^a^	**(n = 350)**
With any adverse events within 30 d	87 (24.9%)
With any procedure-/device-related adverse events within 30 d	4 (1.1%)
With any device deficiency within 30 d	1 (0.3%)
**With any serious adverse events within 30 d**	36 (10.3%)
Cardiac disorders	
** **Atrial fibrillation	1
** **Cardiac arrest	1
** **Cardiac failure congestive	1
** **Tachycardia	1
Gastrointestinal disorders	
** **Abdominal pain	1
** **Lower gastrointestinal hemorrhage	1
** **Pancreatitis	1
General disorders/general physical health deterioration	1
Hepatobiliary disorders/cholelithiasis	1
Infections/pneumonia	1
Injury, poisoning, procedural complications	
** **Fall	1
** **Symptomatic fracture	5
Musculoskeletal disorders	
** **Back pain	14
** **Intervertebral disc degeneration	1
** **Spinal pain	1
Neoplasm/plasma cell myeloma	2
Nervous system disorder/transient ischemic attack	1
Respiratory disorders	
** **Aspiration	1
** **Chronic obstructive pulmonary disease	1
** **Pneumonia aspiration	1^b^
** **Pneumothorax	1
Vascular disorders/vascular stenosis	1

^a^Patients may have had multiple AEs.

^b^Occurred at the end of the procedure and prolonged the subject's hospital stay. The subject was intubated and bronchoscopy was performed. This SAE was considered possibly related to anesthesia and resolved within 2 d.

One subject experienced an SAE of aspiration pneumonia, considered possibly related to anesthesia that occurred at the end of the procedure, prolonging hospital stay. Another subject with a baseline history of coronary artery disease and prior myocardial infarction had a myocardial infarction SAE 105 d postprocedure that was thought possibly related to procedure/anesthesia; symptoms resolved within 2 d with pharmaceutical therapy. All of these device- or procedure-related AEs resolved with proper treatment. All AEs are posted on www.clinicaltrials.gov (NCT01871519).

## DISCUSSION

### Key Results

EVOLVE is the first large prospective on-label as-treated clinical trial designed to include patients commonly seen in clinical practice and to define the efficacy of treatment based on typical parameters as are commonly and currently employed by the CMS LCD guidelines. Statistically significant improvements in each of the 4 coprimary endpoints were demonstrated at 3 mo and at all subsequent time points, and therefore the primary objective of the study was met.

MCIDs are thresholds commonly used to estimate outcome effectiveness.^20^ Improvements at all time points in this study of >5 points exceeded the 1- to 2.5-point threshold for NRS back pain,^20,22^ and ODI improvements >30 points exceeds the 10- to 15-point threshold.^20,21^ Similarly, improvements from baseline in SF-36v2 PCS were >10.5 points, which were greater than the estimated MCID of 3.5 to 4.3 points,^20^ and improvements of >0.3 points exceeded the 0.08 to 0.25 threshold for EQ-5D.^23,24^ It is also important to note that, on average, patients had outcomes within 1 to 3 mo postfracture that were comparable to the estimated prefracture state (Figure 2). In a sensitivity analysis excluding a site receiving consultancy fees, it is important to note that statistically significant improvement in all 4 coprimary endpoints was observed at 3 (the primary objective of the study) and 12 mo, with improvements also greatly exceeding the MCID thresholds described. The findings may simply reflect slightly better results at a single center which is why multicenter trials such as this are more robust.

The secondary endpoints had results similar to the primary endpoints with statistically significant improvements in mean limited activity and bed rest days, kyphotic angulation correction, vertebral height restoration, and the ability to provide self-care as determined by the Barthel Index.

Polypharmacy and side effects from narcotic medications can lead to impaired balance and a subsequent increase in falls in an elderly population;^25^ therefore, reduction in medication usage is important. These data confirm a prominent reduction in the number of patients using opioid analgesics through 12 mo following kyphoplasty.^11,13^

Vertebral deformity correction results observed in this trial were less in comparison to prior BKP RCTs^12,16,18^; however, it is important to note that less baseline deformity was observed, potentially leaving less room for improvement in these parameters. In these prior BKP RCTs,^12,16,18^ mean baseline anterior deformity ranged between 35% and 41% and mean baseline kyphotic deformity at the treated level ranged from –14 to –15º. Baseline anterior deformity in this study was 26%, and baseline kyphotic deformity was –10.5º. In FREE,^12^ 89% of index fractures were Genant grade 3, compared to only 34% in this cohort.

### New Fractures

The rate of additional vertebral fractures observed here (47.6% at 1 yr) was slightly higher than other published rates.^11,16^ By comparison, this study had older patients (average 78.9 yr), had a slightly larger percentage of subjects with multiple prevalent VCFs (61%), and reported less bisphosphonate use (18.6%) compared with, for example, patients in the KAVIAR study (mean age of 75.5 yr, 58% with multiple prevalent VCFs, 58% reporting bisphosphonate usage for those undergoing BKP), which could account for increased predisposition to additional or adjacent VCFs.^16^ Approximately 60% of patients with any subsequent fracture had an adjacent fracture, which is consistent with what is known of VCF natural history.^26^ Papanastassiou et al^27^ analyzed all of the level I and II data on vertebral augmentation and determined that the additional fracture rate for those patients treated with vertebral augmentation was 12% compared with 23% for those patients treated with NSM.

### Safety Assessments

The device-/procedure-related AE rate over 12 mo was 1.4% (5/350) with 4 of these resulting in symptomatic complications (1.14%) that resolved with appropriate treatment. These results are in keeping with previously reported level I and II data on vertebral augmentation reporting a low complication rate.^11-14,18,27,28^

Despite cases of cement extravasation reported here, there were no adverse symptoms associated with displaced cement; this is also consistent with a low rate of symptomatic leakages.^11-14,18,27,28^

### Limitations

Limitations include the fact that this is a nonrandomized open label study. In light of several RCTs recently conducted, sham- or NSM-controlled studies,^11-14,29-32^ in a condition that is so severely painful such as VCF, become exceedingly difficult and could introduce selection bias with the patients having a trending decrease in pain being the only ones who would volunteer for randomization to a sham or NSM group. Controlled studies with sham as a comparator as done previously may now be deemed unethical given the known significant reduction in morbidity and mortality in the surgically treated patients.^1,3,4,6^ Heterogeneity was introduced by including both osteoporotic and neoplastic fractures and by differing sensitivity in imaging modalities for each. Few cancer subjects enrolled but nonetheless, including both was prespecified as the primary analysis, supports generalizability and is representative of patients treated in routine clinical practice. Ninety patients (25%) were lost to follow-up for various reasons prior to 1 yr. Although this rate is not out of the ordinary for a study of this size with a mean age of 78.9 yr, the potential to introduce bias to the statistical analysis remains.

## CONCLUSION

In conclusion, significantly reduced pain and disability, improved function, ambulatory status, self-care abilities, and quality of life following kyphoplasty were observed. Narcotic medications usage was reduced, as were days of bed rest and limited activity. There was statistically significant vertebral deformity correction. Five device-/procedure-related AEs were reported; symptoms of each resolved with appropriate treatment. All cases of cement extravasation were asymptomatic. The rate of new and/or adjacent vertebral fractures after kyphoplasty was relatively high and likely attributed to fracture risk factors such as an older patient population, multiple prevalent VCFs, and less osteoporosis treatment. Improvements in pain, function, and quality of life are prompt and sustained indicating that BKP is rapidly effective and durable up to 1 yr. Statistical significance was attained for all primary endpoints and at all time points. These results support the use of kyphoplasty as a safe and highly effective treatment for painful, acute vertebral body compression fractures in patients commonly referred for treatment with an excellent risk:benefit profile.

### Disclosures

Medtronic Sofamor Danek USA Inc sponsored this study and contributed to study design, data monitoring, statistical analysis, and reporting of results and paid for independent core laboratory and EDC services and the open access license. All authors had complete access to data and were provided all analyses requested. Dr Beall reports grants from Medtronic, during the conduct of the study; however, no compensation was received for data interpretation or manuscript writing; grants and personal fees from Medtronic, nonfinancial support from Amendia, other from Lilly, other from Synthes, other from Johnson and Johnson, grants from Vitacare, grants from Ortho Kinematics, other from DFine, other from Bone Support, other from Convatec, other from Spinal Ventures, grants from Zyga, grants from Liventa, grants, nonfinancial support and other from Vexim, grants from Mesoblast, grants, personal fees, nonfinancial support and other from Vivex, outside the submitted work. Dr Chambers declares the following disclosures: as a study investigator, my institution (University of Alabama at Birmingham) received compensation from Medtronic for study-specific data collection; however, no compensation was received for data interpretation or manuscript writing. Dr Amburgy declares the following disclosures: as a study investigator, my institution (University of Alabama at Birmingham) received compensation from Medtronic for study-specific data collection; however, no compensation was received for data interpretation or manuscript writing. Dr Webb declares the following disclosures: as a study investigator, I received compensation from Medtronic for study-specific data collection; however, no compensation was received for data interpretation or manuscript writing. Dr Goodman declares the following conflicts of interest: as a study investigator, my institution (Alabama Clinical Therapeutics) received compensation from Medtronic for study-specific data collection; however, no compensation was received for data interpretation or manuscript writing; unrelated to this study, I am a consultant for and own stock in Discgenics. Dr Datta declares the following disclosures: As a study investigator, my institution, The BACK Center, received compensation from Medtronic for study-specific data collection; however, no compensation was received for data interpretation or manuscript writing; unrelated to this study, I am a consultant with Spinewave. Dr Easton declares the following disclosures: as a study investigator, my institution (Beaumont Health) received compensation from Medtronic for study-specific data collection; however, no compensation was received for data interpretation or manuscript writing. Dr Linville declares the following disclosures: as a study investigator, I received compensation from Medtronic for study-specific data collection; however, no compensation was received for data interpretation or manuscript writing; unrelated to study I received compensation from Medtronic for consultation services. Dr Talati declares the following disclosures: as a study investigator, my institution (Advanced Diagnostic Imaging, PC) received compensation from Medtronic for study-specific data collection; however, no compensation was received for data interpretation or manuscript writing. Dr Tillman declares the following disclosures: employed as Clinical Program Director by and owns stock and stock options in Medtronic Plc.

## Notes

This work has been presented at the following meetings: American Society of Spine Radiology, San Diego, California, February 22-26, 2017; Society of Interventional Radiology, Washington, District of Columbia, March 6, 2017; The International Society for the Advancement of Spine Surgery, Boca Raton, Florida, April 12-14; 2017; American Association of Neurological Surgeons, Los Angeles, California, April 22, 2017.

## Notes

**Neurosurgery Speaks!** Audio abstracts available for this article at www.neurosurgery-online.com.

## Neurosurgery Speaks (Audio Abstracts)

Listen to audio translations of this paper's abstract into select languages by choosing from one of the selections below.
